# Early biomarkers and potential mediators of ventilation-induced lung injury in very preterm lambs

**DOI:** 10.1186/1465-9921-10-19

**Published:** 2009-03-10

**Authors:** Megan J Wallace, Megan E Probyn, Valerie A Zahra, Kelly Crossley, Timothy J Cole, Peter G Davis, Colin J Morley, Stuart B Hooper

**Affiliations:** 1 Department of Physiology, Monash University, Melbourne, Victoria, Australia; 2 Department of Biochemistry and Molecular Biology, Monash University, Melbourne, Victoria, Australia; 3 Newborn Research, Royal Women's Hospital, Melbourne, Victoria, Australia

## Abstract

**Background:**

Bronchopulmonary dysplasia (BPD) is closely associated with ventilator-induced lung injury (VILI) in very preterm infants. The greatest risk of VILI may be in the immediate period after birth, when the lungs are surfactant deficient, still partially filled with liquid and not uniformly aerated. However, there have been very few studies that have examined this immediate post-birth period and identified the initial injury-related pathways that are activated. We aimed to determine if the early response genes; connective tissue growth factor (*CTGF*), cysteine rich-61 (*CYR61*) and early growth response 1 (*EGR1*), were rapidly induced by VILI in preterm lambs and whether ventilation with different tidal volumes caused different inflammatory cytokine and early response gene expression.

**Methods:**

To identify early markers of VILI, preterm lambs (132 d gestational age; GA, term ~147 d) were resuscitated with an injurious ventilation strategy (V_T _20 mL/kg for 15 min) then gently ventilated (5 mL/kg) for 15, 30, 60 or 120 min (n = 4 in each). To determine if early response genes and inflammatory cytokines were differentially regulated by different ventilation strategies, separate groups of preterm lambs (125 d GA; n = 5 in each) were ventilated from birth with a V_T _of 5 (VG5) or 10 mL/kg (VG10) for 135 minutes. Lung gene expression levels were compared to levels prior to ventilation in age-matched control fetuses.

**Results:**

*CTGF, CYR61 *and *EGR1 *lung mRNA levels were increased ~25, 50 and 120-fold respectively (p < 0.05), within 30 minutes of injurious ventilation. VG5 and VG10 caused significant increases in *CTGF*, *CYR61*, *EGR1*, *IL1-β*, *IL-6 *and *IL-8 *mRNA levels compared to control levels. *CTGF, CYR61, IL-6 *and *IL-8 *expression levels were higher in VG10 than VG5 lambs; although only the *IL-6 *and *CYR61 *mRNA levels reached significance.

**Conclusion:**

*CTGF, CYR61 *and *EGR1 *may be novel early markers of lung injury and mechanical ventilation from birth using relatively low tidal volumes may be less injurious than using higher tidal volumes.

## Introduction

The lungs of very preterm infants have an immature distal airway structure, with a thick air/blood barrier and a small surface area for gas-exchange. They are surfactant deficient because undifferentiated epithelial cells predominate with few type II alveolar cells. As a result, very preterm infants often require respiratory support in the minutes following birth. Although essential for survival, mechanical ventilation of very preterm infants is closely associated with a high risk of developing bronchopulmonary dysplasia (BPD). BPD is characterised by a simplification of airways, a cessation of alveolarisation, hypercellularity, variable fibrosis and capillary dysplasia [1].

Ventilator induced lung injury (VILI) in preterm infants is associated with many different forms of mechanical ventilation [2-7]. The inflammation that results from VILI is thought to play an important role in the pathogenesis of BPD. VILI promotes the recruitment of inflammatory cells such as neutrophils and macrophages and induces many pro-inflammatory cytokines, transcription factors and growth factors leading to abnormal lung development [8,9]. These factors include interleukin (IL)-1β, IL-6, IL-8, IL-10, tumour necrosis factor (TNF)-α, transforming growth factor (TGF)-β_1_, nuclear factor (NF)-κB and interferon-γ [8,10-13]. Although these factors are elevated in response to VILI, a detectable increase can take many hours or days [14], making it difficult to define the initial injury-related pathways involved [9,15]. Identifying the initial injury pathways is critical as the greatest risk of injury may be during the period immediately after birth when the lungs are partially liquid-filled, are surfactant deficient and are not uniformly aerated [16-18]. However, it is unclear whether the above factors are reliable markers of lung injury in studies that are of short duration e.g. investigations of the neonatal resuscitation period.

One of the histological hallmarks of BPD is hypercellularity of the lung [1] and we have recently demonstrated that VILI rapidly stimulates lung cell proliferation in the immature lung [19]. The early response genes connective tissue growth factor (*CTGF*), cysteine-rich 61 (*CYR61*) and early growth response factor 1 (*EGR1*) are known to promote cell proliferation [20,21] and we have recently shown that they are rapidly activated in response to a fetal lung growth stimulus [22]. Previous studies have also demonstrated that these genes are activated in response to lung injury in adults [23-27], but their role in VILI in the preterm neonate is unknown. Thus, our first aim was to investigate whether these early response genes are activated within 15 min-2 h of an injurious insult to the lungs of preterm lambs, before pathological changes to the lung have occurred. To determine their usefulness as early markers of lung injury, we compared their change in expression with changes in the expression of the inflammation genes *IL-1β, IL-6, IL-8 *and *TGF-β*_1_, TNF-α protein levels and NF-κB activity, which have previously been associated with VILI in neonates [8,11,13]. Our second aim was to determine if the mRNA levels of these genes could differentiate between ventilation strategies likely to induce only a mild degree of VILI. To address that aim we determined the mRNA levels of *CTGF, CYR61, EGR1, IL-1β, IL-6 and IL-8 *in preterm lambs resuscitated from birth using tidal volumes of 5 or 10 mL/kg. Based on the known roles of CTGF, CYR61 and EGR1, it is possible that their aberrant expression contributes to abnormal lung development in very preterm infants destined to develop BPD.

## Methods

### Animal experiments

#### Delivery and ventilation of lambs

All experimental procedures on animals were approved by the Monash University Animal Ethics Committee. Pregnant Merino × Border Leicester ewes at 125 or 132 days of gestational age (GA; term is ~147 d) were anaesthetised and the fetal head and neck were exposed for catheterisation and intubation. The fetus was then delivered and ventilated as described below for 135 min. Arterial blood samples were collected every 5 min for the first 15 min and then every 10 min until the end of the experiment. The peak inspiratory pressure (PIP), positive end expiratory pressure (PEEP), mean airway pressure (P_aw_), tidal volume (V_T_), inspiratory and expiratory times, ventilation rate, arterial blood pressure and heart rate were recorded using a data acquisition system (PowerLab, ADInstruments Pty. Ltd., Castle Hill, NSW, Aust.). The alveolar-arterial oxygen difference (AaDO_2_) was calculated using the equation: (P_barometric _- P_H2O_) × FiO_2 _- (PaCO_2_/0.8) - PaO_2_. Control fetuses at the same gestational ages were used to indicate the levels of gene expression prior to ventilation.

#### Time-course for the activation of early response genes caused by injurious ventilation (IV)

Preterm lambs delivered at 132 d gestation (n = 16) were resuscitated and mechanically ventilated from birth using a Dräger "Babylog 8000^+^" (Dräger Medical, Lubeck, Germany). For the first 15 min after birth, lambs were ventilated with an injurious ventilation (IV) protocol, consisting of a tidal volume (V_T_) of 20 mL/kg in the absence of a PEEP. After 15 min, lambs were ventilated using a V_T _of 5 mL/kg and 8 cmH_2_O PEEP for a further 15 (LI 15), 30 (LI 30), 60 (LI 60) or 120 (LI 120) mins (n = 4 for each group).

#### Affect of tidal volume on the activation of early response genes

Preterm lambs delivered at 125 d GA were resuscitated and mechanically ventilated using the Dräger "Babylog 8000^+^" set to deliver a guaranteed V_T _of either 5 (VG5) or 10 (VG10) mL/kg with 8 cmH_2_O of PEEP for 135 min from birth (15 minute resuscitation stabilisation period followed by 2 h of ventilation; n = 5 in each group). The ventilation settings and experimental protocol for these studies have been described previously [28].

#### Post-mortem examination and tissue collection

At the end of each experiment lambs were humanely killed with an overdose of sodium pentobarbitone (i.v.). The lungs were removed, weighed and the left bronchus was ligated. The left lung was cut into small sections and snap frozen in liquid nitrogen for analysis of *CTGF, CYR61, EGR1, IL-1β, IL-6, IL-8 *and *TGF-β*_1 _mRNA levels, active NF-κB levels and TNF-α protein concentrations. The right lung was fixed via the airways, using 4% paraformaldehyde at 20 cmH_2_O for light microscopy.

### Tissue analysis

#### Active NF-κB protein levels

NF-κB protein activity was measured in lung tissue using an electromobility gel-shift assay. Lung nuclear proteins were extracted [29] from lung tissue and the protein concentration was determined using a BioRad *DC *Protein Assay kit (Sigma Aldrich, Australia). Nuclear protein (8 μg) was incubated on ice for 20 min with 2 μl binding buffer (100 mM HEPES, 50 mM MgCl_2_, 50% glycerol, 10 mM EDTA, 500 mM potassium glutamate), 1 μl DTT, 1 μl poly dIdC and 1 μl of a double stranded ^32^P-κB DNA probe containing the cognate κB motif (5'-AGTTGAGGGGACTTTCC-3'; total volume 20 μl). Samples were then electrophoresed for 2 h at 110 V at room temperature in a 5% non-denaturing polyacrylamide (19:1 Acrylamide:Bis-acrylamide) gel with 0.5× TBE buffer. The gel was then dried onto Whatmann 3 mm chromatography paper in a gel drier (Speed Gel SG210D, Savant Instruments, USA) and exposed to a storage phosphor screen for 24 – 48 h at room temperature. The relative levels of active NF-κB bound to the κB motif were quantified by measuring the total integrated density of each band using ImageQuant software (Molecular Dynamics, Sunnyvale, CA). To compare values from different electromobility gel-shift assays, values from each treatment group were expressed as a percentage of the mean value obtained from the same age-matched control fetuses that were run on all blots for the each experiment.

#### TNF-α protein concentration

The concentration of TNF-α in lung tissue was measured using a modified antibody-sandwich method of the enzyme-linked immunosorbent assay [30]. Tissue samples were homogenised in 1× PBS and centrifuged at 2,500 rpm for 20 min. Supernatant, plasma or standards (50 μl) were incubated overnight in a 96-well microtitre plate precoated with 50 μl of TNF-α mouse ascites monoclonal antibody (diluted 1:250 in 3 mM NaN_3_, 20 mM Na_2_CO_3_, 30 mM NaHCO_3_) and blocked with 1% skim milk powder in PBS. Plates were washed five times in PBS with 20% Tween 20 (Wash buffer), then incubated for 2 h with 50 μl of rabbit anti-TNF-α polyclonal antisera (1:500 dilution in 0.001 M PBS/5%BSA). The plates were then washed with buffer and incubated for 1 h with 50 μl of sheep anti-rabbit horseradish peroxidase (diluted 1:1000 in 0.01 M PBS/5% BSA). The plates were then washed, 100 μl tetramethyl benzidine/dimethyl sulphoxide was added and the plates were incubated for 10 – 15 min in the dark before the colour reaction was stopped using 0.5 M sulphuric acid. An automatic plate reader (Original Labsystems Multiskan RC, USA) measured the absorbance (at 450 nm) and the levels of TNFα in each sample were determined by interpolation of the standard curve.

#### TGF-β_1 _gene expression

*TGF-β*_1 _mRNA levels in lung tissue were quantified by Northern Blot analysis as previously described [31]. The total integrated density of the *TGF-β*_1 _mRNA transcript was divided by the total integrated density of the 18S rRNA band for that sample to account for minor differences in total RNA loading between lanes. As a result, the band densities are presented as a ratio of the 18S rRNA band density and, therefore, have no units.

#### Quantitative real-time polymerase chain reaction

*EGR1, CTGF, CYR61, IL-1β, IL-6 *and *IL-8 *mRNA levels in lung tissue were measured using quantitative real-time polymerase chain reaction (qRT-PCR). The primers used for amplification of these genes, the gene accession numbers and the regions amplified are shown in Table 1. Total RNA was extracted, DNase-treated and 1 μg was reverse transcribed into cDNA (M-MLV Reverse Transcriptase, RNase H Minus, Point Mutant Kit; Promega, Madison, WI). qRT-PCR was performed using a Mastercycler^® ^ep gradient S realplex real-time PCR system (Eppendorf, Germany) using 20 μl reactions, containing 1 μl cDNA template (1.5 μg/μl for *IL-6*, 1 μg/μl for *IL-1β, IL-8 *and *CTGF*, 500 ng/μl for *EGR1 *and 200 ng/μl for *CYR61 *and 18S), 1 μl of each forward and reverse primer (10 μM for *IL-1β, IL-6, IL-8, CYR61 *and 18S and 4 μM for *CTGF *and *EGR1*), 10 μl SYBR green (Platinum^® ^SYBR^® ^Green qPCR SuperMix-UDG; Invitrogen Life Technologies, Carlsbad, CA) and 7 μl of nuclease-free water. The thermal profile used to amplify the PCR products included an initial 2 min incubation at 95°C, followed by 35–40 cycles of; denaturation at 95°C for 3 sec, annealing at 59°C (*IL-1β, IL-8 *and *EGR1*) or 60°C (*IL-6, CTGF *and *CYR61*) for 20 sec and elongation at 72°C for 20 sec. The fluorescence was recorded after each 72°C step. Dissociation curves were performed to ensure that a single PCR product had been amplified for each primer pair. Each sample was measured in triplicate and a control sample, containing no template, was included in each run. A threshold value (C_T _value) for each sample was determined. Minor differences in the amount of cDNA template added to each reaction were adjusted by subtracting the C_T _value for 18S from the C_T _value for the gene of interest (ΔC_T_). To enable comparisons between assays, a calibrator sample (in quadruplicate) was run in each assay. The average C_T _value for the calibrator sample was subtracted from the ΔC_T _of each sample (ΔΔC_T_). The mRNA levels of genes of interest were normalized using the equation 2^-ΔΔCT ^and the results were expressed relative to the mean mRNA levels of the gene of interest in non-ventilated control fetuses.

**Table 1:** Primers used for quantitative real-time PCR

**Gene**	**GenBank Accession #**	**Nucleotides amplified**	**Upstream primer 5'-3'**	**Downstream primer 5'-3'**
*EGR1*	DQ239634	444–532	AGGGTCACTGTGGAAGGTC	GCAGCTGAAGTCAAAGGAA
*CTGF*	DQ239672	407–469	TATAGCTCCAGCGACAGCTC	ACGAACTTGACTCAGCCTCA
*CYR61*	DQ239628	286–354	ATCGTCCAAACAACTTCGTG	GGTAACGCGTGTGGAGATAC
*IL-1β*	NM_001009465	353–473	CGATGAGCTTCTGTGTGATG	CTGTGAGAGGAGGTGGAGAG
*IL-6*	NM_001009392	598–705	CGCAAAGGTTATCATCATCC	CCCAGGAACTACCACAATCA
*IL-8*	NM_001009401	438–520	CCTCAGTAAAGATGCCAATGA	TGACAACCCTACACCAGACC
*18S*	X01117	1495–1673	GTCTGTGATGCCCTTAGATGTC	AAGCTTATGACCCGCACTTAC

#### Light microscopy and immunohistochemistry for EGR1 and CYR61

Each lobe of each right lung was cut into 5 mm slices. Every second slice was subdivided into 3 sections and 6 sections were chosen at random from each lobe, cut into ~1 cm × 1 cm sections and embedded in paraffin. Paraffin blocks were randomly selected and 5 μm sections were incubated at 60°C for 2 h, deparaffinised in xylene, rehydrated using graded alcohol washes and washed in PBS and either stained with Haemotoxylin and Eosin (H&E) or treated further for immunohistochemistry. Sections used for immunohistochemistry were then boiled in sodium citrate (0.01 M, pH 6.0) for 20 mins (in a microwave, on high) to enhance antigen retrieval. Sections were then washed in PBS (CYR61 2 × 5 min; EGR1 3 × 5 min) and incubated (CYR61 5 min; EGR1 30 min) in hydrogen peroxide (3%) to block endogenous peroxidase activity. They were then rinsed in water (CYR61 only), washed in PBS and incubated in blocking/permeabilisation buffer (10% normal goat serum and 0.1% TritonX-100 in 0.05 M TrisHCl for CYR61 sections or 25% normal goat serum and 5% BSA in 0.05 M TrisHCl for EGR1 sections) in a humidity chamber (CYR61 30 min; EGR1 45 min, at room temp). The sections were then incubated with the primary antibodies (CYR61 Cat# sc-13100; EGR1 Cat# sc-189, Santa Cruz Biotechnology, California, USA) diluted in DAKO antibody diluent (CYR61, diluted 1:150; EGR1 diluted 1:200) for either 90 min at room temperature (CYR61) or overnight at 4°C (EGR1). Sections were then washed in PBS (0.1% Tween-20) for 5 mins (×3) and incubated with a biotinylated secondary antibody (goat anti-rabbit diluted 1:700; Vector laboratories, Burlingame, CA) in PBS/0.1% Tween 20 (CYR61) or Dako antibody diluent (EGR1) for 1 hour at room temperature. The sections were again washed in PBS (0.1% Tween 20) for 5 mins (×3) before the secondary antibody was detected using the Vectastain ABC detection kit (Vector laboratories). The sections were washed, dehydrated and permanently mounted. Sections that lacked the primary antibodies or the secondary antibody were also included.

Sections were viewed under a light microscope and images were captured at a magnification of 1000× using a digital camera. Analysis was performed on images using ImagePro Plus (Media Cybernetics, MD) on 5 fields of view per section using 3 randomly chosen sections (from different regions of the lungs). For each field of view, the area of tissue positively stained for EGR1 or CYR61 was measured and expressed as a percentage of the total area of tissue. The percentage of stained tissue for each lamb was then averaged for each experimental group. Analysis was performed on the alveolar region of the lung, taking care to avoid areas containing major airways or blood vessels.

#### Data analysis

Data are expressed as the mean ± SEM with the level of statistical significance set at p < 0.05. PaCO_2_, pHa, SaO_2_, FiO_2 _and PIP were analysed using a 2-way repeated measures ANOVA. The immunohistochemistry data was analysed by a nested ANOVA. The relative amounts of active NF-κB (all three bands summed) and the mRNA levels of *TGF-β*_1_, *CTGF, CYR61, EGR1, IL-6, IL-8 *and *IL-1β *were compared between groups using one-way ANOVA. Significant differences indicated by ANOVA were subjected to a least significant difference post-hoc test to identify differences between individual time points and treatment groups.

## Results

### Activation of early response genes following IV

**Figure 1:**
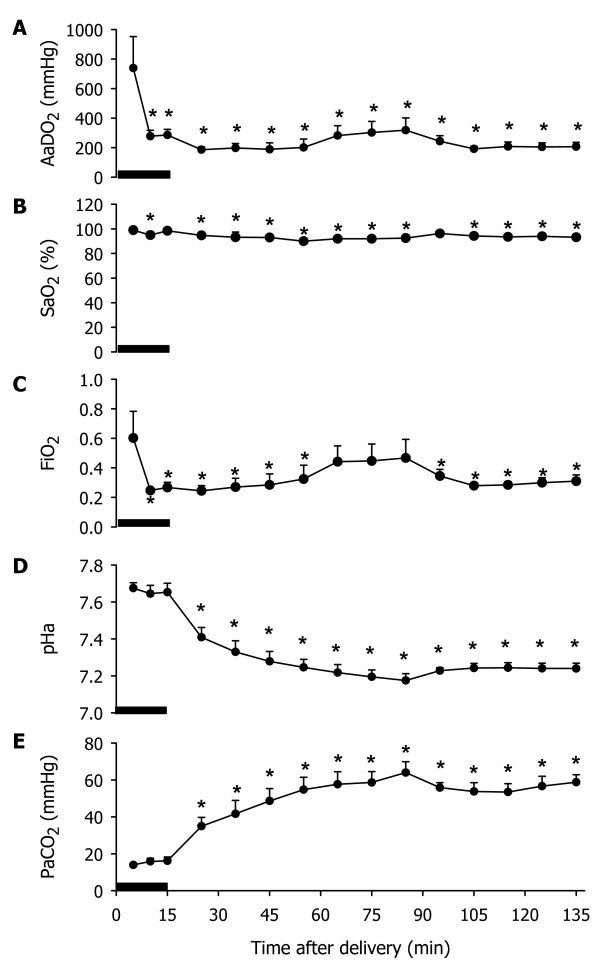
**Blood gas parameters following 15 minutes of injurious ventilation**. The alveolar-arterial difference in oxygenation (AaDO_2_) (**A**), oxygen saturation (SaO_2_) (**B**), fraction of inspired oxygen (FiO_2_) (**C**), arterial pH (pHa) (**D**) and partial pressure of CO_2 _in arterial blood (PaCO_2_) (**E**) in preterm lambs at 132 days of gestation resuscitated at birth using an injurious ventilation strategy then ventilated gently for 120 minutes. Values are mean ± SEM. The black bar indicates 15 min of ventilation with 20 mL/kg V_T _and 0 cmH_2_O of positive end-expiratory pressure. The asterisks (*) represent values significantly different (p < 0.05) to the initial (5 min) time point.

All blood gas and ventilation parameters were similar in the four groups of lambs exposed to 15 mins of IV immediately after birth (LI 15, LI 30, LI 60, LI 120). Thus, only data from the lambs ventilated for 2 hrs after the 15 min IV protocol (LI 120) are presented in Fig 1.

#### Blood gas parameters

Throughout the 135 min experimental period, the SaO_2 _remained at or higher than 95% (Fig. 1). The FiO_2 _was initially reduced from 0.60 ± 0.18 to 0.27 ± 0.03 at the end of the 15 min IV period (V_T _20 mL/Kg, 0 cmH_2_O PEEP), but it was necessary to gradually increase the FiO_2 _to a maximum of 0.47 ± 0.13 at 70 mins after completion of the IV period. The AaDO_2 _was significantly reduced from 739.6 ± 213.1 mmHg to 285.9 ± 38.0 mmHg by the end of the 15 min IV period and then remained at this level for the duration of the experiment. During the 15 min IV period, the PaCO_2 _and pHa remained unchanged at 15 ± 1 mmHg and 7.66 ± 0.02, respectively. However, during the remainder of the experimental period, the PaCO_2 _gradually increased, reaching a maximum of 64 ± 6 mmHg, and the pHa gradually decreased, reaching a minimum of 7.18 ± 0.04 (Fig. 1).

#### Ventilation parameters

During 15 min of IV, the PIP required to administer a V_T _of 20 mL/kg (in the absence of PEEP) decreased (p < 0.02) from 54 ± 2 cmH_2_O at 3 min after birth to 47 ± 3 cmH_2_O by the end of the 15 min IV period. Within 10 min of change in ventilation strategy, the PIP required to deliver a V_T _of 5 mL/kg with 8 cmH_2_O PEEP was reduced (p < 0.001) to 32 ± 1 cmH_2_O. The required PIP did not change further during the remainder of the 120 min ventilation period. However, because of the increasing PaCO_2 _and decreasing pH, it was necessary to gradually increase the ventilation rate from 36.3 ± 6.6 breaths/min at the end of the 15 min IV period to 87.1 ± 18.5 breaths/min at the completion of the experiment. As a result, the mean airway pressure at the end of the 15 min IV period was similar to that at completion of the experiment (15.2 ± 0.5 vs 15.6 ± 0.6 cmH_2_O).

#### Indicators of lung injury

The level of active NF-κB within lung tissue did not significantly change for up to 2 h following 15 min of IV; the levels were similar at 15 (78.2 ± 7.9%), 30 (93.2 ± 27.0%), 60 (109.9 ± 22%) and 120 (70.4 ± 23.3%) min after IV compared with values prior to ventilation measured in age-matched control fetuses (100.0 ± 5.8%). Similarly, TGF-β_1 _mRNA levels in lung tissue were similar at 15 (96.4 ± 2.0%), 30 (99.7 ± 4.2%), 60 (98.3 ± 14.1%) and 120 (99.1 ± 13.6%) minutes after IV, compared with the levels before ventilation in age-matched control fetuses (100.0 ± 3.8%). TNF-α protein levels could not be detected in plasma or tissue homogenates in ventilated lambs or in unventilated age-matched control fetuses.

**Figure 2:**
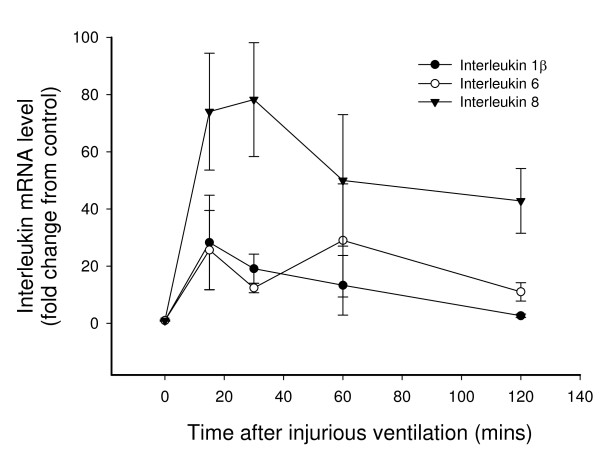
***IL-1β, -6 *and *-8 *mRNA levels following injurious ventilation**. *IL-1β, IL-6 *and *IL-8 *mRNA levels (mean ± SEM) in preterm lamb lungs at 132 days of gestation resuscitated at birth using an injurious ventilation (IV) strategy for 15 minutes, then ventilated gently for 15–120 minutes. Values are expressed as a fold change relative to values in unventilated age-matched control fetuses (T = 0 values). *IL-6 *and *IL-8 *mRNA levels were significantly higher than the levels in unventilated control fetuses (p < 0.05) at all timepoints after the IV period. *IL-1β *mRNA levels were significantly higher than the levels in unventilated control fetuses at 15, 30 and 60 minutes after the IV period.

IV induced a large and sustained increase in *IL-1β, IL-6 *and *IL-8 *mRNA levels; 28.3 ± 16.6, 25.6 ± 13.9 and 74.1 ± 20.4 fold increase respectively (p < 0.05), compared with pre-ventilation control values, within 15 mins of completing IV (Fig 2). Although *IL-1β *mRNA levels had returned to control levels at 120 mins after completion of the IV period, *IL-6 *and *IL-8 *mRNA levels remained significantly elevated (p < 0.05) at 11.0 ± 3.2 and 42.8 ± 11.3 fold, respectively, above pre-ventilation control values at this time (Fig 2).

**Figure 3:**
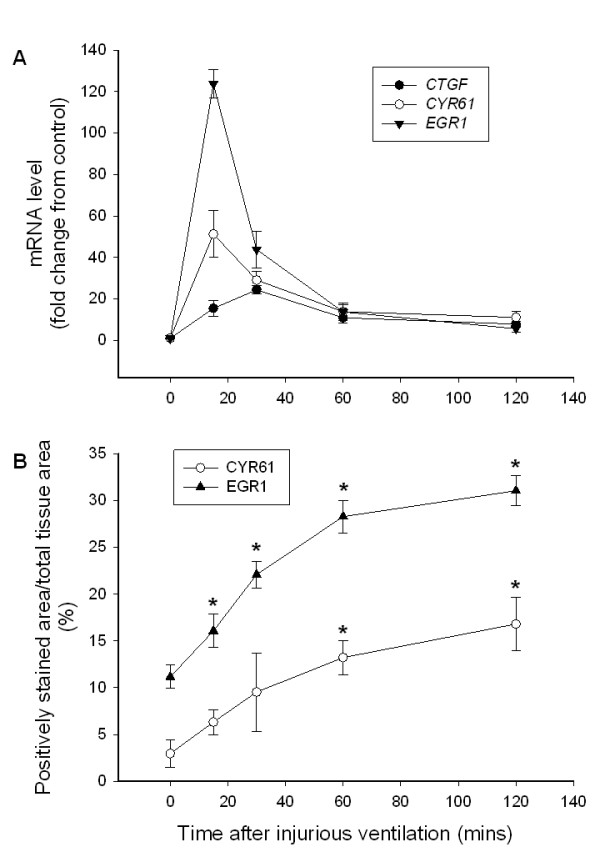
***CTGF*, *CYR61 *and *EGR1 *mRNA levels following injurious ventilation**. (A) *CTGF, CYR61 *and *EGR1 *lung mRNA levels and **(B) **the percentage of tissue staining positive for CYR61 and EGR1 protein in preterm lambs at 132 days of gestation resuscitated at birth using an injurious ventilation (IV) strategy for 15 minutes, then ventilated gently for 15–120 minutes. All values are mean ± SEM and expressed as a fold change relative to values in unventilated age-matched control fetuses (T = 0 values). The mRNA levels of *CTGF, CYR61 *and *EGR1 *were significantly higher (p < 0.05) than the levels prior to ventilation (T = 0), at all time points after IV. The asterisks (*) indicate protein levels of *CYR61 *and *EGR1 *that were significantly higher (p < 0.05) than the levels before ventilation measured in age-matched control fetuses.

IV also induced a time-dependent increase in mRNA levels for *CTGF, EGR1 *and *CYR61*. The expression levels of all three genes were significantly higher (p < 0.05) at every time point after IV, than the pre-ventilation mRNA levels in age-matched control fetuses. *CTGF *mRNA levels increased 15.5 ± 3.8 fold at 15 mins and increased further to 24.4 ± 2.1 fold the control values at 30 mins after the IV period. *CTGF *mRNA levels in lung tissue then declined to 10.9 ± 2.7 fold at 60 mins and to 7.8 ± 1.5 fold of the control values at 120 mins after the IV period (Fig. 3A). Compared with the values prior to ventilation in age-matched control fetuses, *EGR1 *and *CYR61 *mRNA levels increased by 123.7 ± 7.0 and 51.3 ± 11.4 fold, respectively, at 15 mins after the IV period. *EGR1 *and *CYR61 *mRNA levels in lung tissue then declined to 43.9 ± 8.8 and 29.1 ± 4.3 fold above control values at 30 mins, to 13.8 ± 4.1 and 13.7 ± 3.5 fold at 60 mins, and to 11.1 ± 2.7 and 5.6 ± 1.5 fold, respectively, at 120 mins after the IV period (Fig. 3A).

**Figure 4:**
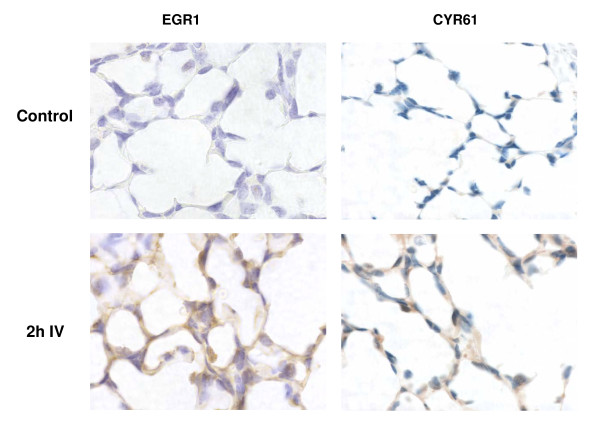
**EGR1 and CYR61 protein levels in lung tissue following injurious ventilation**. Lung tissue sections stained for EGR1 and CYR61 proteins using immunohistochemical techniques. The lung tissue sections shown are representative of the sections from unventilated age-matched control fetuses and preterm lambs at 2 hours after a 15 minute period of injurious ventilation (IV). The brown stain represents lung tissue containing the EGR1 or CYR61 protein. Slides incubated without the primary or secondary antibodies did not show any evidence of brown staining (data not shown).

The increase in *CYR61 *and *EGR1 *gene expression was reflected by a gradual, but marked, increase in the percentage of lung tissue stained positive for these proteins (Fig 3B); representative histological sections immunostained for CYR61 and EGR1 are shown in Figure 4. The percentage of lung tissue labelled positive for the CYR61 and EGR1 proteins increased from 3.0 ± 1.4 and 11.2 ± 1.2% before ventilation in control fetuses to 16.8 ± 2.9 and 31.1 ± 1.6%, respectively (p < 0.05), at 2 hours after IV (Fig. 3B). Sections of lung tissue that lacked the primary antibodies or the secondary antibody showed no evidence of staining. CTGF protein levels could not be determined as none of the commercial antibodies tested recognised ovine CTGF.

### Affect of tidal volume on the activation of early response genes

#### Blood gas and ventilation parameters and indices of lung injury

**Figure 5:**
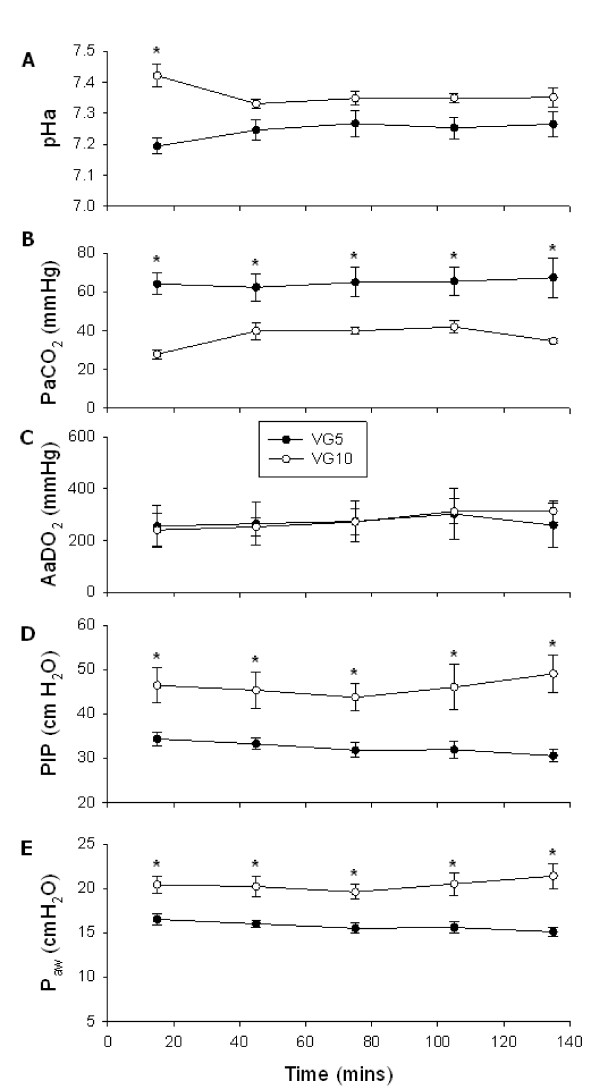
**Blood gas and ventilator parameters during VG5 and VG10 ventilation strategies**. Arterial pH (pHa) (**A**), partial pressure of CO_2 _in arterial blood (PaCO_2_) (**B**), alveolar-arterial oxygen difference (AaDO_2_) (**C**), peak inspiratory pressure (PIP) (**D**) and mean airway pressure (P_aw_) (**E**) in preterm lambs mechanically ventilated from birth at 125 days of gestation. Lambs were mechanically ventilated with either 5 (VG5) or 10 (VG10) mL/kg. Values are mean ± SEM and the asterisks represent values significantly different (p < 0.05) between VG5 and VG10.

**Figure 6:**
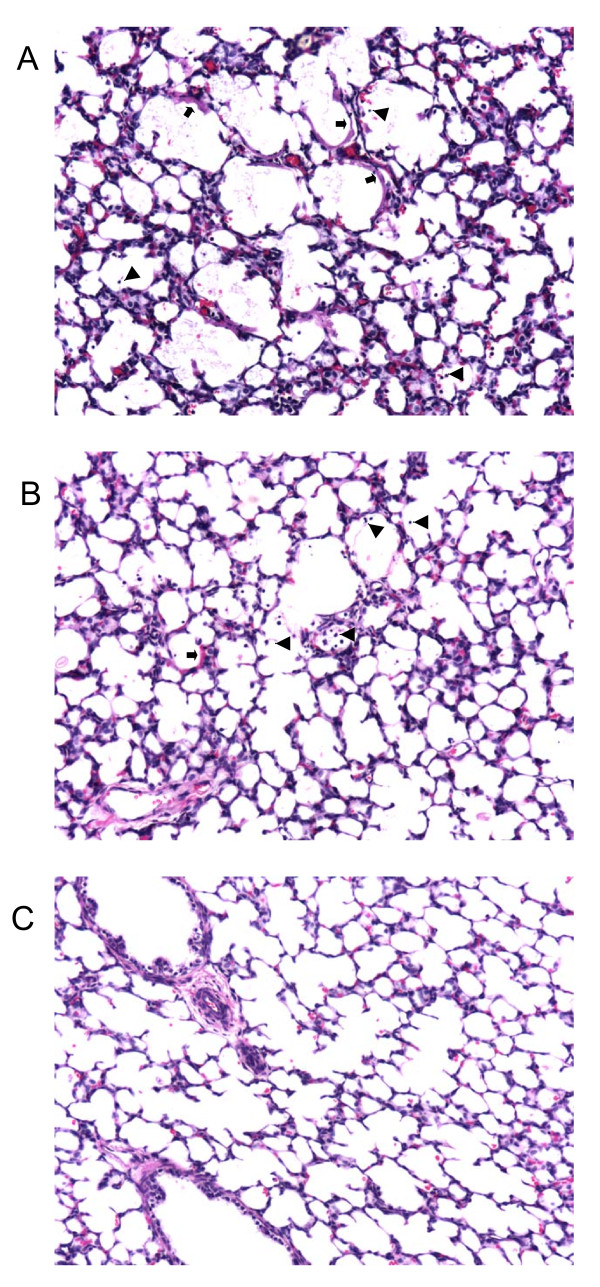
**Histological evidence of lung injury in lambs ventilated with VG5 and VG10 ventilation strategies**. Representative haematoxylin and eosin stained lung tissue sections in preterm lambs mechanically ventilated from birth at 125 d of gestation with a tidal volume of 10 mL/kg **(A) **or 5 mL/kg **(B) **and unventilated control fetuses **(C)**. Hyaline membranes are shown with arrows and detached epithelial cells are shown with arrowheads.

The blood gas and ventilation parameters for these studies have been presented in detail previously [28]. The co-efficient of variation of the delivered V_T _was 6.5 ± 0.3%. The PIP and P_aw _delivered to VG10 lambs was significantly higher (p < 0.05) than the PIP and P_aw _delivered to VG5 lambs throughout the 15 minute resuscitation and 2 h ventilation period (Fig 5). PaCO_2 _values were significantly lower (p < 0.05) in the VG10 group than the VG5 group throughout the 15 minute resuscitation period and 2 h ventilation period. pHa values were significantly higher (p < 0.05) in lambs ventilated at 10 mL/kg compared with lambs ventilated at 5 mL/kg during the resuscitation period but were not different from the 5 mL/kg lambs during the 2 hour ventilation period. The SaO_2 _and AaDO_2 _were similar in both groups (Fig 5). Two of the VG10 lambs developed pneumothoraces and the experiments were terminated (just prior to the planned end of the ventilation period). Subpleural air leaks were also observed in three of the VG10 lambs. None of the VG5 lambs developed pneumothoraces and only one developed a subpleural air leak. At least three H&E stained tissue sections from three different regions of the lung from each lamb were closely examined under the light microscope for evidence of lung injury. All lung tissue sections from lambs ventilated with 10 mL/kg showed substantial and consistent evidence of hyaline membranes, cellular debris and epithelial cell detachment in the bronchioles and terminal airspaces of the lungs (Fig 6). In contrast, there was substantial variation within and between the lungs of the lambs ventilated with 5 mL/kg. Hyaline membranes in VG5 lambs were rare and minor in comparison to VG10 lambs and while epithelial cell detachment was a common finding (Fig 6) in all VG5 lambs, there was substantial regional variation. Hyaline membranes and epithelial cell detachment were not observed in lungs from control fetuses.

#### Indicators of lung inflammation

TNFα protein levels were not detectable and active NF-κB levels and *TGF-β*_1 _mRNA levels within lung tissue were not altered by either of the ventilation procedures (data not shown).

**Figure 7:**
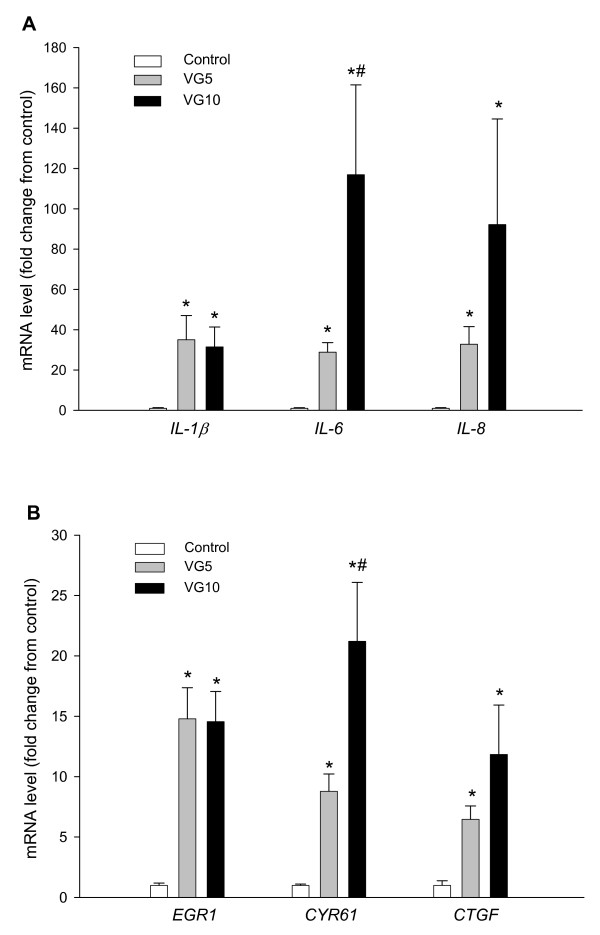
***Interleukin-1β, -6 *and *-8*, *EGR1*, *CYR61 *and *CTGF *mRNA levels in control fetuses and following VG5 and VG10 ventilation strategies**. *IL-1β, IL-6 *and *IL-8 ***(A) **and *EGR1, CYR61 *and *CTGF ***(B) **mRNA levels in unventilated age-matched control fetuses and in preterm lambs mechanically ventilated from birth at 125 days of gestation with either 5 (VG5) or 10 (VG10) mL/kg. The values are mean ± SEM and expressed as a fold-change relative to the mean levels in unventilated control fetuses. The asterisks (*) represents values significantly greater (p < 0.001) than values before ventilation measured in age-matched control fetuses. The hash (#) represents values significantly greater than those in the VG5 lambs (p < 0.05).

The mRNA levels for *IL-1β, IL-6 *and *IL-8 *in lung tissue were significantly increased in both groups of ventilated lambs, compared to the levels prior to ventilation measured in age-matched control fetuses (p < 0.001; Fig 7). The increase in *IL-1β *mRNA levels was similar in VG5 (35.1 ± 12.0 fold) and VG10 (31.5 ± 9.9 fold) lambs and were greater than control levels (1.0 ± 0.3; p < 0.001). However, the increase in *IL-6 *was significantly greater in VG10 (116.9 ± 44.6 fold) lambs compared to VG5 lambs (28.9 ± 4.8 fold, p < 0.05), both of which were significantly higher than the levels before ventilation in control fetuses (1.0 ± 0.3; p < 0.001). The increase in *IL-8 *mRNA levels was also greater in the VG10 lambs (92.2 ± 52.4 fold) than in the VG5 lambs (32.8 ± 8.7 fold) and both groups were significantly higher than control levels (1.0 ± 0.4; p < 0.001), however, due to the large degree of variation between lambs the differences between the two ventilated groups were not statistically significant.

The lung mRNA levels of *EGR1, CYR61 *and *CTGF *were also significantly increased (p < 0.01) in both ventilated groups of lambs, compared to the levels before ventilation in age-matched control fetuses (Fig 7). The fold increase in *EGR1 *mRNA levels relative to control levels (1.0 ± 0.2; p < 0.001) was similar in VG5 (14.8 ± 2.6 fold) and VG10 (14.6 ± 2.5 fold) lambs. The fold increase in *CYR61 *mRNA levels was greater in the VG10 (21.2 ± 4.9 fold; p < 0.01) lambs than in the VG5 treated lambs (8.8 ± 1.4 fold) and both were significantly greater than the levels prior to ventilation in control fetuses (1.0 ± 0.1; p < 0.01). The increase in mRNA levels for CTGF was also greater in the VG10 (11.8 ± 4.1 fold) lambs than in the VG5 treated lambs (6.5 ± 1.1 fold) but the difference between the ventilated groups failed to reach statistical significance. Both groups of ventilated fetuses had significantly higher CTGF mRNA levels than the control fetuses (1.0 ± 0.4; p < 0.001).

## Discussion

Ventilator-induced lung injury (VILI) is closely associated with BPD in very preterm infants [1] and is thought to trigger an inflammatory response which results in abnormal lung development. However, the specific mechanisms by which mechanical ventilation causes lung injury in very preterm infants are largely unknown, as are the pathways resulting in the abnormal lung development that characterise BPD. We have recently demonstrated that VILI in the immature lung induces a rapid increase in distal lung cell proliferation [19] which is consistent with the fibroblast proliferation seen in infants with BPD [1] We have also identified a number of early response genes (*CTGF, EGR1 *and *CYR61*) that regulate cell proliferation and are thought to play a role in normal lung development [22]. As these genes are also involved in adult lung injury and disease [24-27], we investigated their activation following VILI in preterm lambs. We found that *CTGF, EGR1 *and *CYR61 *expression is rapidly increased in a time-dependent manner in response to VILI in very preterm lambs and that *CTGF, CYR61, IL-6 *and *IL-8 *are differentially expressed during high and low tidal volume ventilation strategies. Thus, it is possible that the abnormal lung development that follows VILI, is explained at least in part by the abnormally high expression of these genes. Furthermore, the reduction in pneumothoraces and sub-pleural air-leaks, the histological evidence of lung injury and our gene expression findings indicate that volume-controlled mechanical ventilation (with PEEP) from birth, using a low tidal volume (5 mL/kg) was less injurious than using a tidal volume of 10 mL/kg.

A primary aim of this study was to determine the degree and rapidity of increase in expression of *CTGF, CYR61 *and *EGR1 *following injurious ventilation, in comparison to that of inflammatory factors that have previously been associated with VILI in neonates [8,11,32]. In the present study TNFα protein was not detectable, while NF-κB activity and *TGF-β1 *mRNA levels did not change within 2 hr of VILI, suggesting that these proteins and genes do not form part of the very early response to lung injury in very preterm lambs. In contrast, the increases in *IL-1β, IL-6 *and *IL-8 *after injurious ventilation support the findings of other studies that have also found these inflammatory cytokines are increased at 2–3 h after injurious ventilation from birth [32,33]. Our study extends those findings to demonstrate that *IL-1β, IL-6, IL-8*, *CTGF, CYR61 *and *EGR1 *all responded very rapidly (within 15 minutes of an injurious resuscitation period) and to levels substantially higher (25–125 fold) than those in unventilated controls. These data suggest that the cascade of events leading to lung inflammation and lung remodelling can be rapidly initiated during the immediate resuscitation period after birth. The abnormally high expression levels of these genes was not only limited to resuscitation with high tidal volumes without PEEP, but also occurred in response to ventilation regimens similar to those commonly used for preterm infants.

CYR61 and CTGF are members of the CCN protein family which in mammals consists of 6 proteins (CYR61, CTGF, nephroblastoma-overexpressed1; NOV1 and the Wnt-induced secreted proteins; WISP-1, WISP-2 and WISP-3; [34]). The CCN family are secreted matricellular proteins that form interactions between the extracellular matrix and cell adhesion molecules, leading to diverse cellular responses including cell proliferation, extracellular matrix production, angiogenesis, adhesion, migration, apoptosis and growth arrest [34].

CTGF induces lung fibroblast proliferation, myofibroblast differentiation [35] and the expression of collagen and other extracellular molecules [34]. *CTGF *has increased expression (0.3 fold) in fetal sheep lungs undergoing accelerated lung growth [22] and *CTGF *knockout mice die at birth of respiratory failure due to defects in the rib cage and pulmonary hypoplasia [36]. Although these data indicate that CTGF is important for normal lung growth, abnormally elevated levels of *CTGF *expression are also implicated in the pathogenesis of adult human lung diseases such as idiopathic pulmonary fibrosis [24] and chronic obstructive pulmonary disease [26]. In the adult mouse, bleomycin-induced pulmonary fibrosis [23] and hyperoxia-induced lung injury [25], also exhibit elevated *CTGF *mRNA levels. As fibroblast proliferation, myofibroblast differentiation, hypercellularity and pulmonary fibrosis are commonly associated with VILI in very preterm infants [1] and fetal sheep [19], it is possible that abnormally high CTGF expression following VILI (~25 fold in the current study) may contribute to the pathogenesis of BPD.

CYR61 is structurally and functionally similar to CTGF and also acts as an early response gene. CYR61 acts synergistically with other growth factors to potentiate their mitogenic effects on endothelial, epithelial and fibroblast cells [20,37] as well as to promote collagen and cartilage production [34]. Depending on the cellular milieu, the primary role of CYR61 is thought to be the regulation of angiogenesis by promoting the proliferation of endothelial cells and the production of angiogenic molecules such as vascular endothelial growth factor [38,39]. Interestingly, CYR61 also up-regulates the expression of inflammatory genes, including *IL-1β*, as well as modulators of the extracellular matrix such as proteases and their inhibitors [40]. Similar to *CTGF, CYR61 *expression is also increased (~0.3 fold) in fetal sheep lungs undergoing accelerated growth [22] and abnormally high levels of *CYR61 *have been implicated in the pathogenesis of chronic obstructive pulmonary disease [26] in humans as well as lung injury in adult rodents induced by hyperoxia [25] or volutrauma [27]. Based on its known roles, the large and rapid increase in CYR61 expression (~50 fold in the current study) may contribute to the abnormal lung pathology caused by VILI via several mechanisms. It may contribute to the hypercellularity and fibrosis by directly stimulating the proliferation of fibroblasts and epithelial cells and may upset the normal balance of angiogenic factors, contributing to dysmorphic capillary growth. It may also contribute to the sustained inflammation and abnormal tissue repair that can occur in response to VILI and is an antecedent of BPD in very preterm infants. Our results indicate that increased *CYR61 *expression may play a key role in initiating the cascade of events caused by VILI, as CYR61 protein levels in lung tissue were increased 6-fold within two hours of VILI.

EGR1 is a transcription factor that is rapidly expressed by diverse stimuli that induce growth, differentiation and apoptosis [41]. EGR1 up-regulates the expression of cell cycle regulatory proteins, growth factors, cytokines such as IL-1β, TNFα and TGFβ and other transcription factors including itself and matrix proteins [21,42-45]. *EGR1 *is up-regulated in the fetal sheep during accelerated lung growth [22] and in hemi-pneumonectomy induced compensatory lung growth in adult mice suggesting that it may play a role in regulating normal lung growth [46]. However, *EGR1 *expression is also increased by volutrauma in the adult rat lung [47] and it plays a pivotal role in the response to pulmonary ischaemia-reperfusion injury in the adult mouse [48]. In humans it has been implicated in the pathogenesis of chronic obstructive pulmonary disease [26,49] and vascular pathologies where it can cause vascular lesions, suppress the growth of damaged endothelial cells and modulate vascular tone [reviewed in [43]]. These roles for EGR1, suggest the high levels of its expression induced by VILI (~125 fold in the current study), may contribute to abnormal lung development by its ability to induce cell proliferation, impair vascular development, produce matrix proteins and induce cytokines that promote inflammation.

Regardless of whether CTGF, CYR61 and EGR1 are critical mediators of abnormal lung development caused by VILI, they are likely to be early markers of lung injury. All three genes were very rapidly elevated in response to the injurious ventilation strategy. More importantly, when taken together, the expression levels of *IL-6*, *IL-8*, *CTGF *and *CYR61 *appeared to differentiate between ventilation strategies causing different degrees of lung injury. Expression levels of all four genes were lowest in lambs mechanically ventilated with a tidal volume of 5 mL/kg and were higher in lambs mechanically ventilated with 10 mL/kg that exhibited gross and histological evidence of lung injury. In contrast, *EGR1 *and *IL-1β *appeared not to be sufficiently sensitive to detect any differences between the ventilation strategies. Although the 135 minute ventilation period did not allow time for changes in lung structure to manifest histologically, other evidence indicated that VG10 lambs incurred more lung injury than VG5 lambs. This evidence included the presence of hyaline membranes, detached epithelial cells, red blood cells in the distal lung parenchyma, the presence of blood stained tracheal aspirates, the production of pneumothoraces and subpleural air leaks, and the high PIP required to achieve the tidal volume of 10 mL/kg [28].

## Conclusion

The current international guidelines for neonatal resuscitation (ILCOR) provide little guidance on the most appropriate resuscitation techniques that minimise lung injury in the immediate newborn period when the lungs are partially liquid-filled and not uniformly aerated. Our data indicate that VILI during the immediate newborn period can rapidly (within 15 mins) initiate changes in gene expression which are abnormal and likely to potentiate inflammation and to promote abnormal lung development. Furthermore, our studies indicate that resuscitation and mechanical ventilation at birth with relatively high tidal volumes is potentially more injurious than with relatively low tidal volumes. We also conclude that CTGF, CYR61, EGR1, IL-1β, IL-6 and IL-8 are likely to be useful biomarkers of VILI in the newborn, particularly in studies of short duration.

## Competing interests

The authors declare that they have no competing interests.

## Authors' contributions

MW identified *EGR1, CTGF *and *CYR61 *as likely candidate genes, oversaw the molecular and histological component of the analyses and prepared the manuscript. MP performed the animal experiments and the *TGF-β*_1_, TNF-α and NF-κB analyses. VZ performed the real-time PCR and immunohistochemical analyses. KC supervised the animal experiments and TC provided intellectual input into the studies and provided editorial assistance with the manuscript. CM, PD and SH designed and supervised the animal experiments, obtained funding for the project and provided intellectual input and editorial assistance with the manuscript.
